# Unequal burdens of COVID-19 infection: a nationwide cohort study of COVID-19-related health inequalities in Korea

**DOI:** 10.4178/epih.e2023068

**Published:** 2023-07-31

**Authors:** Jeangeun Jeon, Jieun Park, Min-Hyeok Choi, Hongjo Choi, Myoung-Hee Kim

**Affiliations:** 1 Department of Sociology, Yonsei University, Seoul, Korea; 2 Department of Preventive and Occupational & Environmental Medicine, Pusan National University Medical College, Yangsan, Korea; 3 Office of Public Healthcare Service, Pusan National University Yangsan Hospital, Yangsan, Korea; 4 Department of Preventive Medicine, Konyang University College of Medicine, Daejeon, Korea; 5 Center for Public Health Data Analytics, National Medical Center, Seoul, Korea

**Keywords:** COVID-19, Pandemics, Health inequities

## Abstract

**OBJECTIVES:**

While the Korean government’s response to the coronavirus disease 2019 (COVID-19) pandemic is considered effective given the relatively low mortality rate, issues of inequality have been insufficiently addressed. This study explored COVID-19-related health inequalities in Korea.

**METHODS:**

Age standardization for various health inequality indices was derived using data from the Korean National Health Insurance Service, the Korea Disease Control and Prevention Agency, and the Microdata Integrated Service of Statistics Korea. The slope index of inequality (SII) and relative index of inequality (RII) were calculated for socioeconomic variables, while absolute difference (AD) and relative difference (RD) were used for gender and disability inequalities.

**RESULTS:**

We observed a number of COVID-19-related health outcome inequalities. Gender inequality was particularly noticeable in infection rates, with the rate of women 1.16 times higher than that of men. In contrast, socioeconomic inequality was evident in vaccination rates, with a 4.5-fold (SII, -4.519; 95% confidence interval, -7.403 to -1.634) difference between the highest and lowest household income groups. Regarding clinical progression post-infection, consistent findings indicated higher risk for men (RD for hospitalization, 0.90; severe cases, 0.54; and fatality, 0.65), individuals with disabilities (RD for hospitalization, 2.27; severe cases, 2.29; and fatality, 2.37), and those from lower socioeconomic groups (SII for hospitalization, 1.778; severe cases, 0.089; and fatality, 0.451).

**CONCLUSIONS:**

While the infection risk was nearly ubiquitous, not everyone faced the same level of risk post-infection. To prevent further health inequalities, it is crucial to develop a thoughtful policy acknowledging individual health conditions and resources.

## GRAPHICAL ABSTRACT

[Fig f3-epih-45-e2023068]

## INTRODUCTION

Years have passed since coronavirus disease 2019 (COVID-19) was first identified in 2019. Around the world, governments swiftly implemented strategies to control the transmission of this novel disease. Relative to other nations, the government of Korea opted for less stringent policies in response to COVID-19. Rather than imposing an intensive lockdown, they implemented a milder form of control by encouraging citizens to voluntarily participate in social distancing measures [1]. Despite this, Korea successfully maintained lower rates of COVID-19-related health outcomes and deaths compared to other countries through the end of 2021 [2].

Despite Korea’s relatively successful response to the pandemic, the unequal consequences of this crisis have not been sufficiently addressed. Socioeconomic position (SEP) is a key determinant of health inequalities, supported by numerous studies [3]. COVID-19 is no exception, with a surge of recent studies demonstrating unequal health outcomes related to the virus [4]. However, in Korea, limited research has been conducted on these health outcome inequalities, although a few studies were published in early 2020 [5,6]. As we make transition into the post–COVID-19 era, this important issue must be addressed. Research from various disciplines has indicated an increase in social inequalities in Korea during the pandemic. For instance, unemployment has severely impacted lower-income households and precarious workers [7,8]. The academic performance gap among adolescents has widened based on socioeconomic status [9,10], and discrimination against minorities has increased [11,12]. If social inequality has intensified during the pandemic, health outcomes have likely followed a similar trend. Notably, however, all costs associated with the diagnosis, treatment, and vaccination for COVID-19 were covered by the Korean government, either through general taxation or National Health Insurance. This means that healthcare access was guaranteed for everyone, regardless of their ability to pay, at least for services related to COVID-19. This could potentially reduce socioeconomic inequalities in COVID-19-related health outcomes.

Given the contrasting impacts of socioeconomic factors, this study sought to investigate health inequalities related to COVID-19. These include inequalities in infection rates, vaccination coverage, hospitalization rates, the proportion of critically ill patients, and COVID-19-related mortality rates.

## MATERIALS AND METHODS

### Study setting

This study utilized a customized database from the Korean National Health Insurance Service (KNHIS), which was linked to COVID-19 data from the Korea Disease Control and Prevention Agency (KDCA) and cause-of-death information from the Microdata Integrated Service of Statistics Korea (MDIS). We established a retrospective cohort using the KNHIS customized database. A 10% sample was selected from the entire Korean population who were eligible for the National Health Insurance and Medical Aid program as of January 2020. This selection was made using agestratified and gender-stratified random sampling. We obtained confirmed COVID-19 infection cases and vaccination histories from the KDCA data for the period of October 8, 2020 to April 30, 2022, based on the date of case confirmation. Lastly, by linking the KNHIS data with the cause-of-death statistics from MDIS, we were able to identify deaths attributed to COVID-19 for the period of January 1, 2020 to December 31, 2021.

### Measurements

To estimate the COVID-19 incidence and vaccination rates, we did not consider multiple observations from a single individual. Instead, we created dummy variables to differentiate between samples from individuals who had experienced infection or vaccination at least once and those who had not. We divided vaccination status into 2 categories: those who had received no doses and those who had received at least 1 dose. This was due to the small number of individuals who had received only a single dose of the vaccine (Supplementary Material 1). For those infected with COVID-19, we identified clinical outcomes such as hospitalization; severe cases requiring high-flow oxygen supply, mechanical ventilation, or extracorporeal membrane oxygenation; and death. We used the International Classification of Diseases, 10th revision codes U07.1, U07.2, U10, and U10.9 from the KNHIS database to identify COVID-19-related hospitalizations and severe cases. While the MDIS data allowed us to identify deaths caused by COVID-19 infection, the most recent data available extended only up to December 2021. If we were to calculate the death rate using only these data, it would exclude deaths occurring during the Omicron wave. To account for this longer period including the Omicron wave, we used a proxy variable, selecting death cases from the KNHIS database that occurred within 30 days of infection as COVID-19- related deaths. To verify this approach, we compared fatality rates estimated from the KNHIS data (all-cause death within 30 days of infection) and MDIS data (death caused by COVID-19) for the period from October 8, 2020 to November 30, 2021. We found that these 2 estimates differed by only around 0.01%, which supports the use of a 30-day period without cause of death as a reasonable proxy variable for COVID-19 fatality. We used household income group as a measure of SEP, dividing the cohort into 6 hierarchical groups from the lowest (Medical Aid beneficiaries) to the highest (those in the fifth quintile of health insurance premiums). To identify the effect of the dominant variant, we divided the epidemic period into the first (November 2020 to January 2021), second (November 2021 to January 2022, when the Delta variant was dominant), and third period (February to April 2022, when the Omicron variant was dominant). We chose these 3 distinct eras, each spanning 3 months, because previous studies have varied in their classifications of epidemic periods [13-17].

### Statistical analysis

We estimated age-standardized rates for the 6 household income groups using the direct method. We also determined the slope index of inequality (SII) and the relative index of inequality (RII). These indices measure absolute and relative inequality across multiple groups with a natural hierarchy, considering the population size of each group, per regression methods [18]. We further computed the absolute difference (AD) and relative difference (RD) for the categorical variables of gender and disability. As a sensitivity analysis, we also examined the COVID-19 incidence and fatality rates stratified by the 3 epidemic periods.

### Ethics statement

The study was reviewed and approved by Pusan National University Yansan Hospital Institutional Review Board (04-2022-030). The requirement for written informed consent was waived because the study used public de-identified data.

## RESULTS

Table 1 presents the baseline characteristics of the overall sample and the outcomes related to COVID-19. The sample included a total of 5,196,467 individuals aged 99 years or younger. Of these, 1,642,160 individuals were confirmed to have contracted COVID-19, and 4,368,149 individuals had been vaccinated at least once between October 2020 and April 2022. Among those infected, 21,867 individuals were hospitalized, and an additional 1,329 cases progressed to severe illness. During the same period, 3,819 deaths, occurring within 30 days of infection, were classified as COVID-19-related deaths.

**Figure 1.**
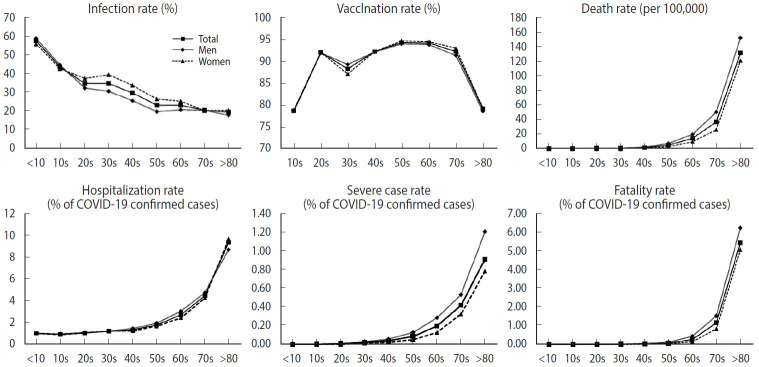
Age-specific rates of coronavirus disease 2019 (COVID-19)-related health outcomes by gender and age. The solid line with the square mark indicates the age-specific rates of COVID-19-related health outcomes for both men and women. The solid line marked with a diamond shape represents the age-specific rates of COVID-19-related health outcomes in men. The dotted line with the triangular mark represents the age-specific rates of COVID-19-related health outcomes in women.

Figure 1 illustrates the age-specific rates of COVID-19 health outcomes, differentiated by gender. Older age groups were more susceptible to hospitalization, progression to severe illness, and death due to COVID-19, while infection was more prevalent among the younger age groups. When considering gender differences, the infection rate graph revealed a disparity in the age group spanning from 30s to 50s, specifically suggesting that women were more frequently infected than men. The differences between men and women were 8.68%, 8.46%, and 6.79% for those in their 30s, 40s, and 50s, respectively. In contrast, for severe cases and deaths, men exhibited slightly higher rates than women, although the extent of this difference was not as pronounced as it was for the infection rate. In the sensitivity analysis, we found that older age groups had a higher fatality rate than younger groups within each epidemic period. We also observed a higher infection rate among middleaged women, with the gap in the infection rate widening during the third period (Supplementary Material 2). The infection rate stratified by period was higher in the low household income group during the first and second periods, but these differences vanished during the third period. However, the lower household income group consistently showed a higher fatality rate across all waves (Supplementary Material 3).

**Figure 2.**
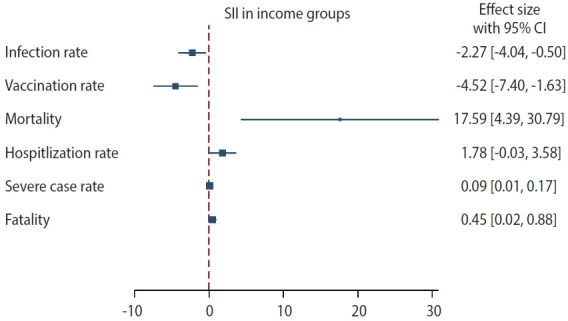
Slope index of inequality (SII) for health outcomes based on household income. The square mark represents the value of the SII for each health outcome, while the horizontal line indicates the 95% confidence interval (CI).

Tables 2 and 3 present the age-standardized rates for COVID-19 infection and associated health outcomes and Figure 2 graphically shows values of the SII for each health outcome according to the household income. These rates were calculated per 100 for all metrics, apart from the mortality rate, which was calculated per 100,000. Both AD and RD are included.

Our findings indicate that individuals from higher SEPs were more frequently infected than lower–SEP groups. The SII and RII values further suggest that the probability of infection decreased by 2.27% (95% confidence interval [CI], -4.04 to -0.50) and 0.93 times (95% CI, 0.62 to 1.39), respectively, as the SEP decreased. The period-stratified analysis revealed that the greatest differences were present among men in the third period (SII, -6.16; 95% CI, -9.91 to -2.30; Supplementary Materrial 4). The vaccination rate was observed to gradually decrease as the household income quintile decreased. The SII estimate of the vaccination rate indicates that the lowest socioeconomic group underwent less vaccination than the highest group by 4.52% (95% CI, -7.40 to -1.63) and 0.95 times (95% CI, 0.84 to 1.07). Socioeconomic inequalities were also evident in hospitalization rates, the proportion of severely ill patients, and fatality rates. Medical Aid beneficiaries, defined as Q0 (the lowest household income group), exhibited the highest rates across all of these indices (Figure 1). The SII and RII estimates corroborated these findings. Based on the RII estimates for hospitalization, severe case rates, and fatality rates, the lowest household income group was more than twice as likely to experience a worsening of clinical conditions once infected than the highest household income group (2.67 times higher for hospitalization, 2.06 times higher for intensive care, and 3.23 times higher for death). These trends were consistently identified in the period-stratified analysis (Supplementary Material 4).

Gender and disability are additional key determinants of health inequalities. All indices captured gender differences. Interestingly, despite having a higher vaccination rate, women exhibited a higher risk of infection than men. However, the gender dynamics became reversed when considering hospitalization, severe cases, and death rates. Regarding disability, no inequality was identified in the infection rate. However, the aftermath of COVID-19 infection proved more severe for disabled individuals. They were more likely to be hospitalized, progress to severe cases, and die from the infection. Compared to non-disabled individuals, the likelihood of disabled individuals progressing to severe illness was more than double across all indices, including hospitalization, severe cases, and fatality rates.

## DISCUSSION

In summary, we noted numerous inequalities in COVID-19-related health outcomes based on SEP, gender, and disability throughout the pandemic. These inequalities were not consistent, but rather varied depending on the specific indicators used. Socioeconomic inequalities were evident in vaccination rates and severe health outcomes, such as hospitalization and mortality rates. However, the infection rate showed a reverse trend, with a higher rate observed in groups with higher household incomes. Gender-based outcomes also varied. While women had a higher risk of infection, men were more susceptible to severe cases. The risk of infection was not notably higher for individuals with disabilities, but these individuals were more likely to experience severe health outcomes once infected compared to those without disabilities.

Several previous studies have similarly explored health inequalities related to COVID-19 in Korea, each focusing separately on the inequality in infection rates or deaths [5,6]. However, most of these studies incorporated health data collected prior to the surge of the Omicron variant, which marked the highest wave of infections in Korea. In this context, our study offers 2 unique contributions. First, it is the first to report a range of health inequality indices, including SII and RII, for COVID-19-related outcomes in Korea. We achieved this by integrating various datasets to cover aspects such as infection, vaccination, progression to severe illness, and death. Second, we extended the study period to include data up to April 2022, encompassing the Omicron wave. By using a 10% sample of the total target population, we enhanced the representativeness of our sample (n= 5,196,467).

During the initial phase of the pandemic, individuals with lower household incomes, such as those covered by the Medical Aid program, experienced higher rates of COVID-19 infection [5,6,19]. However, our results indicate no substantial difference in RII and a slightly higher infection rate among those with higher household incomes, as measured by the SII. The primary distinction between our study and previous research lies in the study period. We included the largest wave of the pandemic, which involved the Omicron variant, while other studies focused on the initial phase of COVID-19. While some studies indicated no significant correlation between household income and COVID-19 infection, most reported positive associations [20]. The lack of clear evidence linking income inequality to infection rates is not easily explained, but several factors should be considered when interpreting these results. First, household income does not fully represent an individual’s SEP. One study found that higher household income was associated with a higher infection rate, but this correlation varied significantly among those with lower levels of education [21]. Another study revealed no significant differences in infection rates based on income inequality, but it did find clear correlations with ethnic disparities [22]. Furthermore, the group with the lowest income (those covered by the Medical Aid program) did not have the lowest infection rate. Instead, they had the third highest infection rate among all 6 income brackets. Therefore, the relationship between income inequality and COVID-19 incidence remains unclear. Further research is needed to better understand the impact of other social determinants and variations in social activities, which could potentially increase transmission opportunities, on infection rates.

In terms of COVID-19 incidence, it is noteworthy that women in their 30s and 40s had a higher rate of COVID-19 infection compared to men and other age groups. A previous review revealed few clinical and epidemiological differences between genders in terms of COVID-19 incidence, although men experienced more severe symptoms and higher fatality rates [23]. One possible explanation for the higher incidence in middle-aged women could be differences in health-seeking behavior between genders [24]. Another factor could be the social and cultural norms, roles, and work environments of women in this age group. A recent study found that jobs with a higher risk of COVID-19 infection were predominantly in the healthcare and welfare sectors, where a large number of women are employed [25]. In the home environment, numerous studies have shown that women have often borne the brunt of childcare responsibilities during the pandemic [26]. Notably, the secondary attack rate of household contacts was highest among those 0-10 years old during the period when the Omicron variant was dominant [27]. The elevated COVID-19 burden among school-aged children could be closely linked to the rise in infection rates among women due to their role in home care. If women in this age group are primarily responsible for childcare, considering the high infection rate among children during the Omicron wave, it is reasonable to suggest that the higher infection rate among women could be attributed to their caregiving roles both at work and at home [28].

The inequity in COVID-19 vaccination is well-documented and linked to individual social determinants of health [29]. A prior study on vaccination trends in Korea indicated that socioeconomic inequalities were largely eliminated due to nationwide vaccination coverage [30]. However, a more recent study revealed ongoing inequality in seasonal influenza vaccination among the elderly, despite full coverage by National Health Insurance [31]. Additionally, lower income levels were correlated with increased vaccination hesitancy, stemming from a lack of confidence and unstable employment status during the early stages of pandemic in 2020 in Korea [32]. Consequently, social, economic, cultural, and institutional barriers to vaccination likely persist in Korea.

Except for COVID-19 incidence, all health indicators reflect gender, disability, and income-related inequality. Theoretically, existing health inequalities and comorbidity could be linked to adverse outcomes of COVID-19 infection, such as hospitalization, severe illness, or death [33]. Previous studies have also suggested that individuals with comorbidities may have a higher mortality rate than those without [34]. However, these comorbidity risks are also socially determined, a concept referred to as the “syndemic of COVID-19, non-communicable diseases, and the social determinants of health” [35]. Our findings may reflect this syndemic of pre-existing health inequality and COVID-19. Furthermore, health and social measures designed to lessen the harmful impact of COVID-19 infection may not be well-structured; as a result [36], the unequal distribution of severity and fatality may have worsened during periods when the Omicron variant was dominant in Korea.

Our study did have certain limitations. First, due to the constraints of our dataset, we included only household income, gender, and disability as social determinants of health. As such, a careful interpretation is necessary, considering the potential impact of unmeasured health determinants. Second, our study did not examine the long-term outcomes of COVID-19 infection due to the relatively brief follow-up period. Future studies should therefore focus on the long-term effects of SEP on COVID-19 infection. Third, we defined a COVID-19 case based on the statutory report of a positive test. If individuals did not undergo testing due to a lack of symptoms or conscious avoidance, they would not be recognized as cases. Unfortunately, we could not account for such effects due to a lack of testing information. Fourth, the insurance premium for employment-based National Health Insurance is determined by the earned income of employed individuals, while the premium for area-based insurance or qualification for the Medical Aid program is determined by household income and assets. If a household has multiple employed members, their insurance premiums would differ based on their individual earned incomes, not household income. Therefore, our operational definition of household income may not accurately reflect the actual situation. Finally, as our study was designed as an individual-level cohort study, we did not fully consider regional-level inequity, the impact of health and social measures, or macro-level determinants such as policy, governance, and politics. These factors should be considered in future studies.

We recommend that social policy be shaped with more nuance, considering the varying risk levels among different groups of people. Health inequalities are not merely a straightforward result of the pandemic. Instead, they may suggest that our approach did not adequately address pre-existing social inequality. In a pandemic situation, the risk of infection is nearly universal across the population. However, not everyone who becomes infected faces the same level of risk. Some individuals, once infected, are particularly susceptible to fatal outcomes. Therefore, an effective policy should extend beyond simply providing free healthcare. Korea needs a prudent policy that recognizes the unique health conditions and resources of each individual to prevent further health inequalities.

## Figures and Tables

**Figure f3-epih-45-e2023068:**
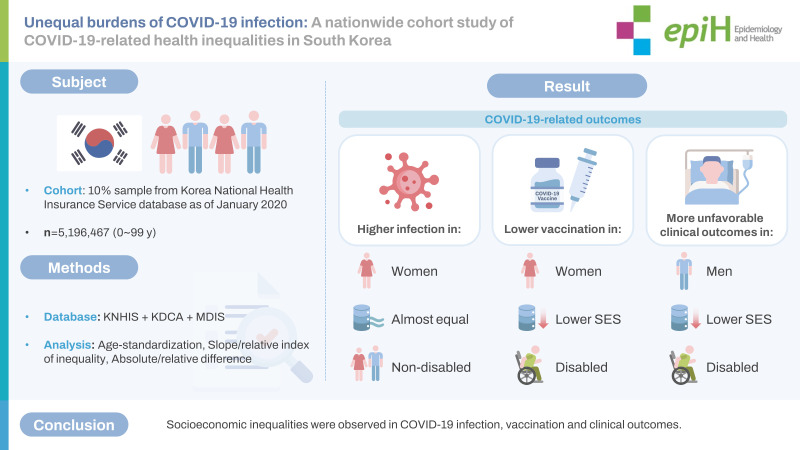


**Table 1.** Baseline characteristics of the total sample and COVID-19-related health outcomes^1^

Characteristics		No. of total sample (n=5,196,467)	p-value^2^	No. of confirmed COVID-19 cases (n=1,641,264)	p-value^2^	No. of individuals with at least 1 dose of vaccination (n=4,368,149)	p-value^2^	No. of COVID-19 cases with hospitalization (n=21,867)	p-value^2^	No. of severe COVID-19 cases (n=1,329)	p-value^2^	No. of deaths caused by COVID-19 (n=3,819)	p-value^2^
Gender			<0.001		<0.001		<0.001		0.001		<0.001		0.756
	Men	2,598,923 (50.0)		769,898 (46.9)		2,181,551 (49.9)		10,013 (45.8)		746 (56.1)		1,801 (47.2)	
	Women	2,597,544 (50.0)		871,366 (53.1)		2,186,598 (50.1)		11,854 (54.2)		583 (43.9)		2,018 (52.8)	
Age (yr)			<0.001		<0.001		<0.001		<0.001		<0.001		<0.001
	<10	362,456 (7.0)		210,172 (12.8)		0 (0.0)		1,231 (5.6)		2 (0.1)		0 (0.0)	
	10-19	475,731 (9.1)		209,396 (12.8)		374,187 (8.6)		1,050 (4.8)		3 (0.2)		1 (0.0)	
	20-29	687,733 (13.2)		242,769 (14.8)		632,708 (14.5)		1,565 (7.2)		16 (1.2)		8 (0.2)	
	30-39	701,092 (13.5)		247,690 (15.1)		618,659 (14.2)		2,020 (9.2)		48 (3.6)		22 (0.6)	
	40-49	833,784 (16.0)		250,481 (15.3)		768,622 (17.6)		2,340 (10.7)		87 (6.5)		54 (1.4)	
	50-59	875,295 (16.8)		204,566 (12.5)		825,722 (18.9)		2,883 (13.2)		167 (12.6)		160 (4.2)	
	60-69	679,421 (13.1)		158,073 (9.6)		639,899 (14.6)		3,705 (16.9)		306 (23.0)		435 (11.4)	
	70-79	371,603 (7.1)		76,629 (4.7)		342,839 (7.8)		3,218 (14.7)		321 (24.1)		887 (23.2)	
	≥80	209,352 (4.0)		41,488 (2.5)		165,513 (3.8)		3,855 (17.6)		379 (28.5)		2,252 (59.0)	
Household income			<0.001		<0.001		<0.001		<0.001		<0.001		<0.001
	Q5 (highest)	1,430,114 (27.5)		463,701 (28.2)		1,197,852 (27.4)		5,933 (27.1)		376 (28.3)		1,108 (29.0)	
	Q4	1,109,570 (21.3)		373,832 (22.8)		903,043 (20.7)		4,200 (19.2)		243 (18.3)		617 (16.2)	
	Q3	918,277 (17.7)		287,723 (17.5)		779,009 (17.8)		3,508 (16.0)		184 (13.8)		464 (12.1)	
	Q2	727,554 (14.0)		225,780 (13.8)		636,078 (14.6)		2,749 (12.6)		184 (13.8)		385 (10.1)	
	Q1 (lowest)	861,245 (16.6)		248,817 (15.2)		730,182 (16.7)		3,623 (16.6)		211 (15.9)		656 (17.2)	
	Q0 (Medical Aid)	149,707 (2.9)		41,411 (2.5)		121,985 (2.8)		1,854 (8.5)		131 (9.9)		589 (15.4)	
Type of medical insurance			<0.001		<0.001		<0.001		<0.001		<0.001		<0.001
	Medical Aid	149,707 (2.9)		41,411 (2.5)		121,985 (2.8)		1,854 (8.5)		131 (9.9)		589 (15.4)	
	Employment-based health insurance	3,624,070 (69.7)		1,244,087 (75.8)		3,075,080 (70.4)		13,781 (63.0)		724 (54.5)		2,074 (54.3)	
	Area-based health insurance	1,422,690 (27.4)		355,766 (21.9)		1,171,084 (26.8)		6,232 (28.5)		474 (35.7)		1,156 (30.3)	
Residential area			<0.001		<0.001		<0.001		<0.001		<0.001		<0.001
	Metropolitan	2,187,109 (42.1)		703,694 (42.9)		1,842,838 (42.2)		9,745 (44.6)		667 (50.2)		1,565 (41.0)	
	Urban	2,571,761 (49.5)		818,923 (49.9)		2,151,011 (49.2)		10,064 (46.0)		556 (41.8)		1,645 (43.1)	
	Rural	437,597 (8.4)		118,647 (7.2)		374,300 (8.6)		2,058 (9.4)		106 (8.0)		609 (15.9)	
Disability			<0.001		<0.001		<0.001		<0.001		<0.001		<0.001
	Disabled	272,053 (5.2)		67,121 (4.1)		234,860 (5.4)		3,478 (15.9)		304 (22.9)		1,183 (31.0)	
	Non-disabled	4,924,414 (94.8)		1,574,143 (95.9)		4,133,289 (94.6)		18,389 (84.1)		1,025 (77.1)		2,636 (69.0)	

Values are presented as number (%). COVID-19, coronavirus disease 2019; Q, quartile.

1 Data period used in analysis: October 8, 2020 to April 30, 2022.

2 Using the chi-square test.

**Table 2.** Age-standardized rates of COVID-19-related health outcomes among the general population, with absolute and relative differences across socioeconomic groups^1^

Characteristics		Infection rate (%)	Vaccination rate (%)	Mortality rate (per 100,000)
Total rate (95% CI)		31.81 (31.76, 31.86)	83.44 (83.37, 83.52)	10.44 (9.54, 11.34)
Gender				
	Men	29.54 (29.48, 29.61)	83.37 (83.26, 83.48)	13.35 (11.74, 14.97)
	Women	34.15 (34.07, 34.22)	83.49 (83.37, 83.60)	8.14 (7.12, 9.16)
	*(i) Absolute diff.^2^*	4.60	0.12	-5.21
	*(ii) Relative diff.^2^*	1.16	1.00	0.61
Household income				
	Q5 (highest)	32.77 (32.68, 32.87)	84.28 (84.13, 84.44)	7.90 (6.56, 9.24)
	Q4	32.50 (32.39, 32.61)	83.62 (83.44, 83.79)	9.17 (7.15, 11.19)
	Q3	31.27 (31.15, 31.39)	83.54 (83.35, 83.73)	9.32 (7.00, 11.63)
	Q2	31.65 (31.51, 31.78)	83.91 (83.70, 84.13)	11.77 (8.69, 14.85)
	Q1 (lowest)	29.92 (29.80, 30.04)	81.86 (81.67, 82.05)	12.68 (10.16, 15.20)
	Q0 (Medical-Aid)	31.74 (31.38, 32.11)	79.41 (78.86, 79.95)	28.55 (21.61, 35.49)
	*(i) SII (95% CI)*	-2.271 (-4.044, -0.498)	-4.519 (-7.403, -1.634)	17.590 (4.388, 30.792)
	*(ii) RII (95% CI)*	0.931 (0.623, 1.391)	0.949 (0.841, 1.071)	4.624 (2.036, 10.506)
Disability				
	Disabled	30.89 (30.54, 31.25)	80.96 (80.49, 81.42)	21.98 (17.84, 26.12)
	Non-disabled	31.80 (31.75, 31.85)	83.61 (83.53, 83.69)	9.01 (8.10, 9.93)
	*(i) Absolute diff.^2^*	-0.90	-2.66	12.96
	*(ii) Relative diff.^2^*	0.97	0.97	2.44

COVID-19, coronavirus disease 2019; CI, confidence interval; Q, quartile; SII, slope index of inequality; RII, relative index of inequality; diff., difference; MDIS, Microdata Integrated Service of Statistics Korea; KNHIS, Korean National Health Insurance Service.

1 Data period used in the analysis of infection and vaccination rate: October 8, 2020 to April 30, 2022; Mortality rate was estimated using MDIS cause-of-death data with a denominator of the whole population, including non-infected individuals; Since the available periods of KNHIS and MDIS data differed, an adjustment was made for the analyzed period; Finally, data from January 2021 to December 2021 were used to calculate mortality rate.

2 Reference group for absolute and relative diff.: men (gender), non-disabled (disability).

**Table 3.** Age-standardized rates of health outcomes among those infected with COVID-19, with absolute and relative differences across socioeconomic groups^1^

Characteristics		% of COVID-19 confirmed cases		
		Hospitalization rate	Severe case rate	Fatality rate
Total rate (95% CI)		1.63 (1.61, 1.65)	0.11 (0.11, 0.12)	0.34 (0.33, 0.36)
Gender				
	Men	1.72 (1.68, 1.75)	0.15 (0.14, 0.17)	0.43 (0.41, 0.45)
	Women	1.55 (1.52, 1.58)	0.08 (0.08, 0.09)	0.28 (0.27, 0.30)
	*(i) Absolute diff.^2^*	-0.17	-0.07	-0.15
	*(ii) Relative diff.^2^*	0.90	0.54	0.65
Household income				
	Q5 (highest)	1.46 (1.42, 1.50)	0.10 (0.09, 0.11)	0.28 (0.26, 0.30)
	Q4	1.52 (1.47, 1.57)	0.11 (0.10, 0.12)	0.34 (0.31, 0.36)
	Q3	1.64 (1.58, 1.70)	0.11 (0.09, 0.13)	0.33 (0.30, 0.36)
	Q2	1.60 (1.54, 1.67)	0.14 (0.11, 0.16)	0.35 (0.32, 0.39)
	Q1 (lowest)	1.69 (1.63, 1.75)	0.11 (0.09, 0.12)	0.36 (0.33, 0.39)
	Q0 (Medical-Aid)	3.82 (3.62, 4.02)	0.21 (0.17, 0.25)	0.88 (0.80, 0.96)
	*(i) SII (95% CI)*	1.778 (-0.029, 3.584)	0.089 (0.008, 0.170)	0.451 (0.018, 0.884)
	*(ii) RII (95% CI)*	2.672 (0.321, 22.225)	2.064 (0.001, 6,650.850)	3.228 (0.030, 347.999)
Disability				
	Disabled	3.42 (3.27, 3.57)	0.23 (0.20, 0.27)	0.71 (0.66, 0.76)
	Non-disabled	1.51 (1.48, 1.53)	0.10 (0.10, 0.11)	0.30 (0.29, 0.31)
	*(i) Absolute diff.^2^*	1.91	0.13	0.41
	*(ii) Relative diff.^2^*	2.27	2.29	2.37

COVID-19, coronavirus disease 2019; CI, confidence interval; Q, quartile; SII, slope index of inequality; RII, relative index of inequality; diff., difference.

1 Data period used in the analysis: October 8, 2020 to April 30, 2022.

2 Reference group for absolute and relative diff.: men (gender), non-disabled (disability).

## SUPPLEMENTARY MATERIALS

Supplementary Material 1. Age-specific number of COVID-19 vaccination by gender

Supplementary Material 2. Age and gender specific infection rate and fatality by the epidemic curves.

Supplementary Material 3. Household income and gender specific infection rate and fatality by the epidemic curves.

Supplementary Material 4. Age standardized rates of COVID-19 infection and fatality rate equality by gender and the epidemic waves
