# Supplementary material for: Unequal burdens of COVID-19 infection: a nationwide cohort study of COVID-19-related health inequalities in Korea
Source: Epidemiol Health. 2023 Jul 31;45:e2023068. doi: 10.4178/epih.e2023068 (PMC10667578; doi:10.4178/epih.e2023068)
Supplement: Supplementary Material 1. — Age-specific number of COVID-19 vaccination by gender [file epih-45-e2023068-Supplementary-1.docx]

Supplementary Material 1. Age-specific number of COVID-19 vaccination by gender

| Age | Men (%) | | | Women (%) | | |
| --- | --- | --- | --- | --- | --- | --- |
|  | 0 | 1 dose | 2 doses | 0 | 1 dose | 2 doses |
| <10 | 185840 (7.15) | 0 (0.00) | 0 (0.00) | 176616 (6.8) | 0 (0.00) | 0 (0.00) |
| 10-19 | 52912 (2.04) | 5153 (0.2) | 188039 (7.24) | 48632 (1.87) | 4398 (0.17) | 176597 (6.8) |
| 20-29 | 29112 (1.12) | 4416 (0.17) | 327188 (12.59) | 25913 (1) | 3739 (0.14) | 297365 (11.45) |
| 30-39 | 39428 (1.52) | 7408 (0.29) | 321324 (12.36) | 43005 (1.66) | 6042 (0.23) | 283885 (10.93) |
| 40-49 | 33369 (1.28) | 4330 (0.17) | 386782 (14.88) | 31793 (1.22) | 3812 (0.15) | 373698 (14.39) |
| 50-59 | 26426 (1.02) | 2832 (0.11) | 411885 (15.85) | 23147 (0.89) | 2944 (0.11) | 408061 (15.71) |
| 60-69 | 20534 (0.79) | 1982 (0.08) | 311030 (11.97) | 18988 (0.73) | 2142 (0.08) | 324745 (12.5) |
| 70-79 | 14552 (0.56) | 1076 (0.04) | 152509 (5.87) | 14212 (0.55) | 1311 (0.05) | 187943 (7.24) |
| >=80 | 15199 (0.58) | 950 (0.04) | 54647 (2.1) | 28640 (1.1) | 2173 (0.08) | 107743 (4.15) |
| Total | 417372 (16.06) | 28147 (1.08) | 2153404 (82.86) | 410946 (15.82) | 26561 (1.02) | 2160037 (83.16) |
