# Supplementary material for: Unequal burdens of COVID-19 infection: a nationwide cohort study of COVID-19-related health inequalities in Korea
Source: Epidemiol Health. 2023 Jul 31;45:e2023068. doi: 10.4178/epih.e2023068 (PMC10667578; doi:10.4178/epih.e2023068)
Supplement: Supplementary Material 2. — Age and gender specific infection rate and fatality by the epidemic curves. [file epih-45-e2023068-Supplementary-2.docx]

Supplementary Material 2. Age and gender specific infection rate and fatality by the epidemic curves.


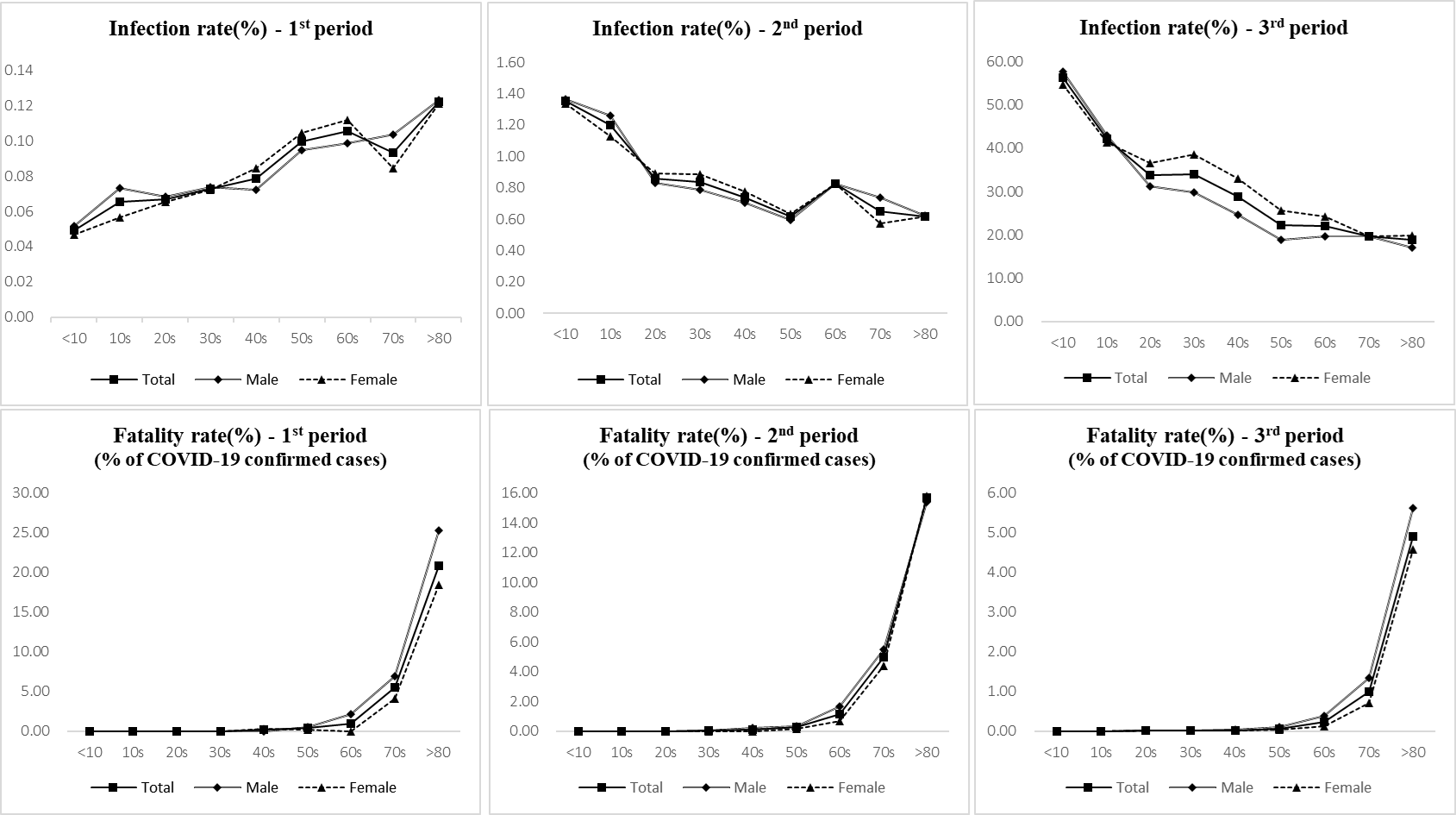


* The classification of the period is as follows: the 1st period (2020.11-2021.01), the 2nd period, delta dominant (2021.11-2022.01), and the 3rd period, omicron dominant (2022.02-2022.04).
