# Supplementary material for: Unequal burdens of COVID-19 infection: a nationwide cohort study of COVID-19-related health inequalities in Korea
Source: Epidemiol Health. 2023 Jul 31;45:e2023068. doi: 10.4178/epih.e2023068 (PMC10667578; doi:10.4178/epih.e2023068)
Supplement: Supplementary Material 4. — Age standardized rates of COVID-19 infection and fatality rate equality by gender and the epidemic waves [file epih-45-e2023068-Supplementary-4.docx]

Supplementary Material 4. Age standardized rates of COVID-19 infection and fatality rate equality by gender and the epidemic waves

|  |  |  | 1^st^ period | | 2^nd^ period | | 3^rd^ period | |
| --- | --- | --- | --- | --- | --- | --- | --- | --- |
|  |  |  | Men | Women | Men | Women | Men | Women |
|  |  |  | (95% CI) | (95% CI) | (95% CI) | (95% CI) | (95% CI) | (95% CI) |
| Infection rate | Household income | (i). SII (95% CI) | 0.032  (-0.011, 0.074) | 0.029  (0.013, 0.044) | 0.009  (-0.127, 0.145) | 0.114  (-0.016, 0.245) | -6.157  (-9.910, -2.403) | 0.027  (-1.605, 1.658) |
|  |  | (ii). RII (95% CI) | 1.447  (0.000, 19446.065) | 1.385  (0.000, 15607.393) | 1.011  (0.051, 19.929) | 1.143  (0.059, 21.962) | 0.803  (0.519, 1.242) | 1.001  (0.675, 1.485) |
|  | Disability | (i). Absolute Diff.* | 0.02 | 0.03 | -0.02 | -0.08 | 0.45 | -0.99 |
|  |  | (ii.) Relative Diff.* | 0.01 | 0.01 | 0.01 | 0.01 | 0.01 | 0.01 |
| Fatality rate | Household income | (i). SII (95% CI) | 0.773  (-0.689, 2.234) | 1.903  (0.484, 3.322) | 1.236  (0.140, 2.331) | 1.352  (0.413, 2.290) | 0.524  (-0.044, 1.093) | 0.330  (0.066, 0.595) |
|  |  | (ii). RII (95% CI) | 1.701  (0.162, 17.896) | 3.410  (0.359, 32.359) | 3.014  (0.187, 48.618) | 2.745  (0.239, 31.551) | 4.523  (0.025, 815.580) | 2.598  (0.022, 304.661) |
|  | Disability | (i). Absolute Diff.* | 1.42 | 1.21 | 1.11 | 1.52 | 0.43 | 0.26 |
|  |  | (ii.) Relative Diff.* | 0.02 | 0.02 | 0.02 | 0.02 | 0.03 | 0.02 |

* Reference group for absolute and relative differences: Non-disabled (Disability). The classification of the period is as follows: the 1st period (2020.11-2021.01), the 2nd period, delta dominant (2021.11-2022.01), and the 3rd period, omicron dominant (2022.02-2022.04). SII=slope inequality index, RII=relative inequality index, CI=confidence interval.
